# Updated Core Competencies for Disaster Medicine and Public Health

**DOI:** 10.1001/jamanetworkopen.2025.60176

**Published:** 2026-02-20

**Authors:** Rita V. Burke, Janine Cadet, Norma A. Quintanilla, Lauren Knieser, Thomas D. Kirsch, Nikolas I. Wada, Larissa Unruh, Kaitlin Rainwater-Lovett, Jeffrey D. Freeman

**Affiliations:** 1 Department of Population and Public Health Sciences Keck School of Medicine, University of Southern California, Los Angeles; 2 Department of Emergency Medicine, Keck School of Medicine, University of Southern California, Los Angeles; 3 National Center for Disaster Medicine and Public Health, Uniformed Services University of the Health Sciences, Bethesda, Maryland; 4 Henry M. Jackson Foundation for the Advancement of Military Medicine, Inc, Bethesda, Maryland; 5 PointClickCare, Baltimore, Maryland

## Abstract

**Importance:**

In 2012, the National Center for Disaster Medicine and Public Health (NCDMPH) created a set of core competencies for disaster medicine and public health through an expert stakeholder panel. The COVID-19 pandemic and other significant events have reshaped experts’ understanding of which core competencies should be prioritized to ensure that health professionals are prepared for disasters and public health emergencies.

**Objective:**

To determine whether the original core competencies should be updated given the many advancements in the field over the last decade.

**Design, Setting, and Participants:**

For this mixed-methods qualitative study, in April 2024, the NCDMPH convened 20 subject matter experts in clinical medicine, emergency management, government, and academia to provide qualitative feedback surrounding the current set of 11 disaster health core competencies.

**Main Outcomes and Measures:**

A Delphi survey incorporating the potential changes or additions to the initial core competencies was distributed to 42 additional stakeholders between April and July 2024 to quantify the level of agreement. Gwet AC2, a measure of interrater agreement, was applied to evaluate the strength of agreement for the need to change each core competency. Competencies with substantial or near-perfect agreement were updated.

**Results:**

Twenty subject matter experts proposed 23 changes to the original core competencies in an initial focus group. Sixteen respondents who completed all items of the Delphi survey reached near-perfect agreement on updating 1 competency and associated subcompetency. The original competency required a demonstration of knowledge of personal safety measures that can be implemented in a disaster or public health emergency. This competency was refined as the identification of physical, psychological, and security hazards associated with the incident. A subcompetency was added that included explaining physical and psychological health, safety, and security risks associated with disasters and public health emergencies.

**Conclusions and Relevance:**

In this mixed-methods evaluation, subject matter experts agreed that 1 of 11 disaster health core competencies required modification. These changes included refined language on recognizing hazards and explaining safety and security risks, reflecting observations and experiences during the COVID-19 pandemic and recognition of diverse types of health. These changes ensure training and education of disaster health responders remains synchronized with developments in the preparedness and response communities.

## Key Points

**Question:**

Based on expert consensus, what changes should be made to the original Core Competencies for Disaster Medicine and Public Health?

**Findings:**

In this mixed-methods qualitative study, 2 of the 23 proposed competency changes showed near-perfect interrater agreement, suggesting these competencies require updating. The updates in the competencies focus on the identification of hazards and explaining the associated risks in disaster settings.

**Meaning:**

Implementing the changes recommended through this study will align diverse educational needs within the disaster medicine and public health sectors and, ultimately, create more effective and well-prepared responses.

## Teaser

This qualitative study reports the results of an expert consensus survey process to update the National Center for Disaster Medicine and Public Health’s list of core competencies for health professionals in disasters and public health emergencies.

## Introduction

Effective national disaster preparedness integrates competencies across health specialties and professions. Diverse teams are required to address all aspects of the disaster management cycle, and competencies form the foundation from which a field can create additional, more specialized training or a professional can enhance their current skills. Without a national accreditation process or standards, disaster health core competencies serve as essential skills and concepts, establish baseline expectations, and ensure consistent application of knowledge in practice.^1^

In 2010, the American Medical Association convened a group of stakeholders to develop a set of core competencies for disaster medicine and public health (DMPH) preparedness. The Core Competencies for DMPH were published in 2012 and aimed to establish a unified knowledge base for those expected to contribute to disaster or public health emergency response.^2^ These competencies included knowledge of preparedness, response plans, situational awareness of hazards and personal safety, clinical and public health management of diverse populations, ethical and legal principles, and recovery considerations (Table). The original core competencies have been used in a wide array of curricula developed to train individuals within disaster roles among medical and public health practitioners who carry out Emergency Support Function #8.^3^ Some of these organizations include the Medical Reserve Corps, emergency medical services, and varying nursing academic curricula.^4,5^ Additionally, the competencies have guided the creation of specialized core curricula in fields such as disaster nursing, mental health, and pediatrics.^5,6^ These trainings have standardized the practicing workforce in this sector; therefore, future updates to the core competencies will impact both current personnel and future generations.

**Table.** Core Competency Changes Proposed by the Subject Matter Expert Focus Group

Original core competency	Proposed change
Competency 1.0: Demonstrate Personal and Family Preparedness for Disasters and Public Health Emergencies	No proposed change
Competency 2.0: Demonstrate knowledge of one’s expected role(s) in organizational and community response plans activated during a disaster or public health emergency	C2: Understand how the medical and public health systems are structured on the federal, state and local levels.
	C2.1A: Explain one’s role within the incident command system in a disaster or public health emergency.
	C2.1B: No change to 2.0 or 2.1
Competency 3.0: Demonstrate situational awareness of actual/potential health hazards before, during and after disaster or public health emergency	No proposed change
Competency 4.0: Communicate effectively with others in a disaster or public health emergency	C4: Learn about various communication systems to effectively engage with others during a disaster or public health emergency.
	C4.1: Identify credible sources for information in a disaster or public health emergency.
	C4.3: Understand strategies for sharing information during disasters or public health emergencies.
	C4.4: Identify cultural opportunities and challenges in the development and dissemination of risk communication during a disaster or public health emergency.
Competency 5.0: Demonstrate knowledge of personal safety measures that can be implemented in a disaster or public health emergency	C5.0A: Identify hazards associated with the incident, including physical, psychological, and security risks in a disaster or public health emergency.^a^
	C5.0B: No change
	C5.1: Explain general physical and psychological health, safety and security risks associated with disasters and public health emergencies.^b^
	C5.2: Describe risk reduction measures that can be implemented to mitigate or prevent illness or injury
Competency 6.0: Demonstrate knowledge of surge capacity assets, consistent with one’s role in organizational, agency, and/or community response plans	C6.0: Demonstrate knowledge of the individual elements of surge capacity (eg, staff, supplies, space, systems) in accordance with one’s role in the organization, agency, and community response.
	C6.2: Identify strategies for surge management to enhance health and public health capacity and capability.
Competency 7.0: Demonstrate knowledge of principles and practices for the clinical management of all ages and populations affected by disasters and public health emergencies in accordance with the professional scope of practice	C7.0: Understand and apply the principles and practices relevant to clinical providers responding to disasters and public health emergencies, in line with their professional scope of practice.
	C7.2: Explain methods for rationing health care services and apply them in disaster situations.
Competency 8.0: Demonstrate knowledge of public health principles and practices for the management of all ages and populations affected by disasters and public health emergencies	C8.2: Combine 8.2 and 8.3 8.2: Identify all ages and population with functional and access needs who may be more vulnerable to adverse health effects in a disaster of public health emergency. 8.3: Identify strategies to address functional and access needs to mitigate adverse health effects of disaster and public health emergencies.
	C8.4: Describe effective deployment principles and practices to safeguard the health of all ages and communities impacted by a disaster or public health emergency.
Competency 9.0: Demonstrate knowledge of ethical principles to protect the health and safety of all ages, populations and communities affected by a disaster or public health emergency	C9: Add new Sub-Competency: Equitable distribution of services and materials within marginalized groups during and after disasters and public health emergencies.
	C9.2: Combine 9.2 and 9.3 9.2: Describe ethical issues and challenges associated with crisis standards of care in a disaster or public health emergency. 9.3: Describe ethical issues and challenges associated with allocation of scarce resources implemented in a disaster or public health emergency.
Competency 10.0: Demonstrate knowledge of legal principles to protect the health and safety of all ages, populations, and communities affected by a disaster or public health emergency	C10.1: Identify the various levels of government issues likely to arise during disasters and public health emergencies.
	C10.3: Describe the legal issues and challenges, including awareness of policies related to health and the public health response.
Competency 11.0: Demonstrate knowledge of short and long-term considerations for recovery of all ages, populations and communities affected by a disaster or public health emergency	C11.4: Discuss the strategies used to monitor the mental and physical health impacts of disasters and public health emergencies on responders and their families.

^a^ Competency C5.0A was accepted as a refinement of competency 5.0.

^b^ In competency C5.1, the words “physical and psychological” were accepted as an addition to the existing language.

Due in part to lessons learned from the COVID-19 pandemic, the past decade has produced significant growth in our understanding of DMPH,^7^ as well as substantial changes in our civilian and military health systems.^8^ The increasing frequency and intensity of extreme weather events present significant threats to human health, infrastructure, and the environment, often triggering or exacerbating other natural disasters.^9^ These occurrences suggest an evolving need for adaptation and preparedness, emphasizing the crucial role of developing core competencies that incorporate new knowledge to effectively prepare the disaster response workforce for the future threat environment. This study leveraged multidisciplinary subject matter expertise to apply lessons learned and update the 2012 DMPH Core Competencies. These updated competencies can provide more effective tools for the ever-changing field of disaster health response.

## Methods

### Subject Population and Recruitment

This qualitative study was conducted to update core competencies based on a 2-phased, mixed-methods design involving subject matter experts (SMEs). The SMEs were chosen based on their professional backgrounds, areas of expertise, and organizational knowledge. Individuals who assisted with development of the original core competencies^8^ and were instructors of the Disaster Health Core Curriculum were also invited as SMEs to participate in the focus groups. The researchers (R.V.B., N.A.Q., L.K., and T.D.K.) contacted potential SMEs via email starting in October 2022, and outreach ceased in March 2023 once a minimum of 20 SMEs had been secured with even distribution across the 4 SME categories. For the Delphi survey, researchers contacted potential survey participants via email beginning in April 2024 through July 2024.

The SME categories represented 4 main fields: clinical medicine and public health, emergency management and medical systems, government, and academia. Clinical SMEs were professionals with medical or nursing degrees, including doctorates, who held distinguished positions in domestic health care systems and hospitals or actively conducted research in emergency preparedness. Their experience included national and international lectures on preparedness and disaster medicine, leadership roles in hospital departments, and medical officers for Disaster Medical Assistance Teams within the National Disaster Medical System of the US Department of Health and Human Services (HHS). Academic SMEs were university faculty members specializing in emergency medicine, health care ethics, infectious diseases, or disaster preparedness and response at accredited medical or public health schools. Emergency medical system SMEs were certified emergency managers holding advanced degrees who served as current or former Chief Disaster or Operating Officers or Deputy Commissioners in Emergency Management in major US cities; led multisectoral partnerships and coalitions; and specialized in earthquake preparedness, exercise development, and emergency management higher education. Government SMEs included experienced federal professionals, medical officers, and public health practitioners from HHS agencies (eg, US Centers for Disease Control and Prevention, US Public Health Service, Health Resources and Services Administration) or a state Governor’s Office of Emergency Services. Their expertise spanned national and international health security policy, strategic planning for public health emergency preparedness, and clinical field operations.

The qualitative research component consisted of a focus group with SMEs, while the quantitative component used the Delphi method to analyze the data produced by the focus group. This research received an exempt study determination by the Human Research Protections Program at the Uniformed Services University as the information collected presented low risk to participants and is subject to the provisions of the National Defense Authorization Act 21, Section 716, which provides an exemption from the requirements of 44 USC §§ 3506(c), 3507, and 3508 for the voluntary collection of information during the conduct of research and program evaluations that are conducted or sponsored by the Uniformed Services University and funded through the Defense Health Program. This study is consistent with the Standards for Reporting Qualitative Research (SRQR) reporting guideline (eTable in Supplement 1).^10^ The Stat59 Delphi module ensured that SMEs provided informed consent to the Delphi process.^11^ The Stat59 consent was provided at the first encounter with the Delphi online survey, and SMEs were required to acknowledge the consent before proceeding with the Delphi survey.

### Expert Focus Group

The National Center for DMPH (NCDMPH) convened 20 SMEs to provide feedback on the current core competencies in April 2024. This focus group initially met as a large group to discuss the evolution of DMPH over the past decade and how the competencies can better reflect these changes. Facilitators were experienced senior researchers in disaster health response who practice clinical medicine and nursing and previously led the development of the disaster health core competencies in 2012.^2^ After the large group meeting, the focus group was divided based on expertise, and each small group was assigned 2 to 3 original competencies to review and propose changes. Participants were prompted to review and edit the current disaster core competencies by drawing from their experiences over the last decade, particularly during the COVID-19 pandemic. Data were analyzed using Altas.ti, version 23.2.1 (Scientific Software Development GmbH) by coding into multiple themes based on the original competencies and any newly suggested competencies.

### Delphi Survey

A survey containing the proposed changes to the competencies was sent between April and July 2024 via the Stat59 Delphi module to the 42 SMEs who provided informed consent following recruitment to gather their insights and level of agreement. Participants rated their agreements with proposed changes using a 7-point Likert scale (strongly disagree [1] to strongly agree [7]) and provided additional comments as desired.^12^

### Data Analysis

To evaluate whether changes to the competencies were required, the original 7-point Likert scale was collapsed into a 3-point scale (scores of 1, 2, and 3 were coded as 1; 4 was coded as 2; and 5, 6, and 7 were coded as 3). This scale better reflected the ultimate purpose of the survey, which was to determine whether experts disagreed (1), were unsure (2), or agreed (3) with the proposed changes. We assessed agreement across all survey respondents by calculating Gwet AC2, a measure of interrater reliability.^13^ Gwet AC2 can be applied to individual items as well as the entire set of responses and tends to be more robust to differences in the underlying frequency distribution than other measures.^14^ Gwet AC2 represents interrater agreement as a score between −1 (no agreement) and +1 (perfect agreement). For each proposed change, Gwet AC2 was calculated and 95% CIs were estimated via bootstrapping. All data analysis was performed between February and April 2025 using R, version 4.4.2 (R Foundation for Statistical Computing). Although there is no formal rule for defining levels of agreement, we used interpretation guidelines suggested by Gwet.^15^

## Results

This study evaluated updates to DMPH core competencies that serve as the basis for disaster health training and education. Using feedback collected during an April 2024 focus group of experts in clinical medicine, emergency management, government, and academia, a Likert-scale survey was developed, and the results guided the selection of DMPH core competency changes.

### Expert Focus Group

Following the focus group, Competency 1 (Personal and Family Preparedness) and Competency 3 (Situational Awareness) were the only 2 competencies that did not have proposed modifications as an outcome. The final 23 proposed competency changes are enumerated in the Table.

### Consensus Reports

**Figure 1.**
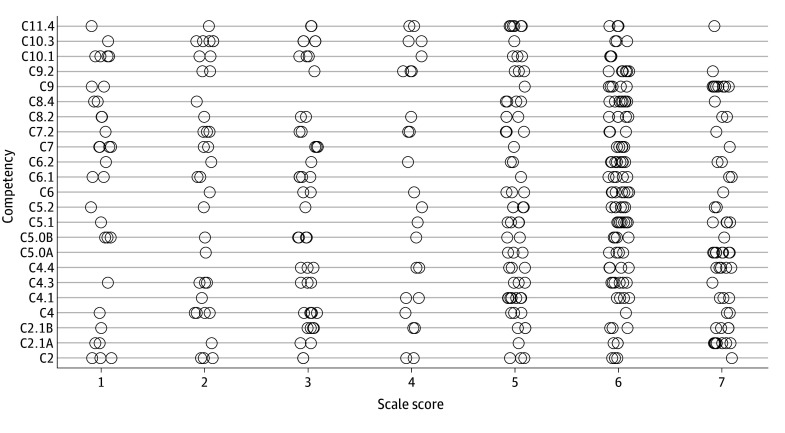
Distribution of Survey Responses by Proposed Competency Scores on the x-axis correspond to the survey’s 7-point Likert scale (from strongly disagree [1] to strongly agree [7]). Circles represent individual responses.

A total of 42 SMEs were invited to participate in the Delphi study. Seventeen experts participated in the survey, and 16 (38.1%) rated all proposed changes. Responses in the original 7-point Likert scale are displayed in Figure 1; the overall median response was 5, and 16 (70%) of the proposed changes had a median rating above 4. After collapsing scores into a 3-point scale, the overall median rating was 3 (range, 1-3), and 16 competencies (70%) had a median rating of 3.

**Figure 2.**
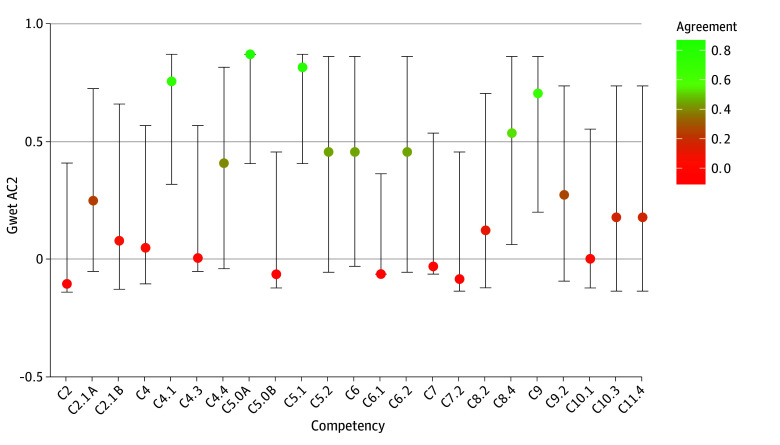
Gwet AC2 Agreement Scores With 95% CIs Gwet AC2 is a statistical measure designed to assess interrater reliability, applicable to various data scales including nominal, ordinal, interval, and ratio. The estimates for each individual competency change are shown. Whiskers represent 95% CIs.

The interrater agreement estimates across all responses was 0.25 (95% CI, 0.08-0.42), which corresponds to fair agreement across raters, according to guidelines suggested by Gwet.^15^ The estimates for each individual competency change are displayed in Figure 2. Due to the single round of the Delphi survey and moderate participation in the survey, we applied a high threshold to implement changes to the competencies. Of the 23 proposed changes to the competencies, 2 changes (C5.0A and C5.1) were met with near-perfect agreement among survey respondents, indicating the need to adopt these modifications.

The changes primarily focused on refining the language describing the competencies and subcompetencies to ensure greater clarity and precision. The original core competency 5.0 required individuals to demonstrate knowledge of personal safety measures that can be implemented in a disaster or public health emergency. This competency was modified to require individuals to identify hazards associated with the incident, including physical, psychological, and security risks in a disaster or public health emergency. Subcompetency 5.1 was refined to add the phrase “physical and psychological,” such that one should be able to explain physical and psychological health, safety, and security risks associated with disasters and public health emergencies.

Near-perfect agreement among survey respondents occurred for proposed changes to competency 5.0 and the associated subcompetency 5.1 (Table). A significant change was proposed for competency 5.0 that motivates situational awareness and risk assessment in emergency situations. Similarly, the modification to subcompetency 5.1 acknowledges the potential for both physical and psychological harm during emergencies and highlights the need for comprehensive risk management strategies.

Overall, the results of the analysis suggest that the proposed changes to the competencies and subcompetencies were generally well received by participants. The modifications that were met with near-perfect agreement represent important refinements to the language and scope of the competencies, reflecting current best practices within DMPH.

## Discussion

This study convened DMPH experts from across the US to determine whether the original core competencies should be updated in light of the events occurring over the last decade. The expert focus group discussed core competency updates in a large group and within specialty-specific breakout groups, followed by a survey of this group and additional SMEs to determine which, if any, of the proposed changes achieved expert consensus. Although survey respondents showed strong agreement on some competency changes, the proposed changes reflect a wide range of challenges faced by professionals in DMPH. Sufficiently addressing all of these challenges likely requires a specialty-specific approach geared toward the educational needs of the major groups and subgroups of responders.

The definition of consensus used in published Delphi studies spans a wide spectrum.^16^ We attempted to be as precise as possible in the definitions to permit transparency and alternative interpretation. Sixteen of the 23 changes received a median score of 3, indicating general agreement with the proposed competency changes. Of these, we chose to update the one competency and one subcompetency showing near-perfect (C5.0A and C5.1) interrater agreement. While the threshold for updating the competencies was strict, the next 2 competencies with strong Gwet AC2 agreement (C4.1 and C9.0) each had experts that disagreed or strongly disagreed with implementing the language changes, suggesting additional edits or discussion may be needed to ensure these competencies adequately reflect needs across disaster health fields.

Some important themes emerged from the expert working group. In particular, the focus group engaged in a comprehensive discussion on differences in subpopulations that create vulnerabilities in disaster management. The updates to one competency and one subcompetency also align with the first priority of the Sendai Framework from the United Nations Office for Disaster Risk Reduction, which focuses on disaster risk understanding across the dimensions of capacity, exposure, hazard characteristics, and vulnerability.^17^ Individuals perceive hazards and interpret the associated risks differently,^18^ but morbidity and mortality can be reduced if risks are identified and communicated effectively.^19^ Reducing vulnerabilities through the identification of hazards and understanding of risks are now reflected in the updates; however, the addition of a subcompetency (C9 in the Table) on equitable distribution of resources did not have similarly strong interrater agreement, suggesting additional work is needed to appropriately hone this language for inclusion.

The current landscape of competencies within DMPH reveals a significant gap: a lack of uniformity across a DMPH workforce. This inconsistency stems from the fact that, historically, competencies have been siloed within individual specialties or targeted professions involved in the field. The field of disaster medicine has core competencies that are primarily intended for physicians in emergency medicine residency programs.^20^ Similarly, disaster nursing competencies are intended for these specific types of clinicians.^21^ Meanwhile, the field of public health has many competencies^22^ but without a clear set focused on disaster public health. This has led to differences in education, training, and best practices among the multidisciplinary personnel who participate in disaster and emergency planning, response, or recovery. Revising the competencies was born out of a need to bridge this gap, and the refinements described here aim to more explicitly define what is required as a foundation for disaster health professionals. Instead of demonstrating knowledge of risks to health, the changes require one to identify specific risks and relate these to explicit types of health, namely physical and psychological. We seek to establish a standardized baseline for all professionals operating in this field, and increased precision in the foundational core competency language represents a significant step toward focusing education and training curricula in DMPH.

The implications of the updated competency and subcompetency are far reaching. In educational and professional development settings, they can serve as a blueprint for standardized curricula planning and the creation of specialty-specific lessons. Furthermore, they provide a valuable roadmap for professionals seeking to enhance their own competence and pursue professional development opportunities related to disaster response. This, in turn, will enhance the overall effectiveness and coordination of efforts in responding to and recovering from future emergencies and times of crisis.

### Limitations

This study had the potential for group effect bias and was limited by a small sample size. We aimed to select expert stakeholders with geographical and workforce variability and representation. While there were 20 participants in the focus group, it is possible that group effect bias contributed to the proposed changes. Forty-two stakeholders were invited to participate in the survey, but only 16 completed all questions. This smaller participant pool likely contributed to greater variance in the Likert scale scores, which made reaching consensus challenging. This was supported by asymmetric bootstrapped 95% CIs around point estimates for several competency changes, likely because of skewed rating distributions. Moving forward, we recognize the need to address the survey challenges posed by time constraints and expert availability, as these limited us to a single iteration of the survey. To enhance future studies and improve generalizability, larger sample sizes and multiple survey iterations will be used.

## Conclusions

The fields of DMPH are evolving significantly, reflecting the changing nature of disasters and public health emergencies. Continuously assessing and improving the core competencies is crucial to remain synchronized with developments in the disaster response community. By proactively implementing updates reached by expert consensus, we can better align the educational needs of the DMPH sectors. This approach will ultimately lead to a more effective and well-prepared response to the challenges we may face in the future.

## Notes

Supplement 1. **eTable.** Alignment of Study Methods with the Standards for Reporting Qualitative Research

Supplement 2. Data Sharing Statement
