# Supplementary material for: Updated Core Competencies for Disaster Medicine and Public Health
Source: JAMA Netw Open. 2026 Feb 20;9(2):e2560176. doi: 10.1001/jamanetworkopen.2025.60176 (PMC12924103; doi:10.1001/jamanetworkopen.2025.60176)
Supplement: Supplement 1. — eTable. Alignment of Study Methods with the Standards for Reporting Qualitative Research [file jamanetwopen-e2560176-s001.pdf]

Supplemental Online Content

Burke RV, Cadet J, Quintanilla NA, et al. Updated core competencies for disaster management and public health. *JAMA Netw Open*. 2026;9(2):e2560176. doi:10.1001/jamanetworkopen.2025.60176

eTable. Alignment of Study Methods with the Standards for Reporting Qualitative Research

This supplemental material has been provided by the authors to give readers additional information about their work.

© 2026 Burke RV et al. JAMA Network Open.

eTable. Alignment of Study Methods with the Standards for Reporting Qualitative Research[1]

Item Number	Item Topic	Line Location
**Title and abstract**		
S1	Title	1
S2	Abstract	40-81
**Introduction**		
S3	Problem formulation	82-111
S4	Purpose or research question	111-114
**Methods**		
S5	Qualitative approach and research paradigm	120-125, 149-151
S6	Researcher characteristics and reflexivity	124-126, 162-164
S7	Context	128-147
S8	Sampling strategy	122-127
S9	Ethical issues pertaining to human subjects	152-156
S10	Data collection methods	158-169, 172-175
S11	Data collection instruments and technologies	168, 182, 187
S12	Units of study	172-175
S13	Data processing	177-181
S14	Data analysis	181-187
S15	Techniques to enhance trustworthiness	187-188
**Results/findings**		
S16	Synthesis and interpretation	191-237
S17	Links to empirical data	Table 1, Figure 1
**Discussion**		
S18	Integration with prior work, implications, transferability, and contribution(s) to the field	240-297, 313-318
S19	Limitations	299-310
**Other**		
S20	Conflicts of interest	323
S21	Funding	326-333

[1] O'Brien BC, Harris IB, Beckman TJ, Reed DA, Cook DA. Standards for reporting qualitative research: a synthesis of recommendations. Acad Med. 2014;89(9):1245-1251.

© 2026 Burke RV et al. JAMA Network Open.
