# Supplementary material for: Updated Core Competencies for Disaster Medicine and Public Health
Source: JAMA Netw Open. 2026 Feb 20;9(2):e2560176. doi: 10.1001/jamanetworkopen.2025.60176 (PMC12924103; doi:10.1001/jamanetworkopen.2025.60176)
Supplement: Supplement 2. — Data Sharing Statement [file jamanetwopen-e2560176-s002.pdf]

Data Sharing Statement

Burke. Updated Core Competencies for Disaster Management and Public Health. *JAMA Netw Open*. Published February 20, 2026. doi:10.1001/jamanetworkopen.2025.60176

Data
Data available: No

Additional Information
Explanation for why data not available: Our data is de-identified, and requests to access the data can be emailed to ncdmph@usuhs.edu
